# Prevention of Localized Osteitis in Mandibular Third-Molar Sites Using Platelet-Rich Fibrin

**DOI:** 10.1155/2013/875380

**Published:** 2013-04-04

**Authors:** Donald R. Hoaglin, Gary K. Lines

**Affiliations:** Arizona Center for Implant, Facial and Oral Surgery, 18301 N 79th Avenue, Building G, Suite 185, Glendale, AZ 85308, USA

## Abstract

*Purpose*. To review our experience utilizing platelet rich fibrin (PRF), which is reported to aid in wound healing of extraction sites, for the prevention of localized osteitis following lower third-molar removal. *Materials and Methods*. PRF was placed in the mandibular third-molar extraction sites, 200 sites total, on 100 consecutive patients treated in our practice, by the authors. The patients were managed with standard surgical techniques, intraoperative IV antibiotic/steroid coverage, and routine postoperative narcotic analgesics/short-term steroid coverage. All patients were reevaluated for localized osteitis within 7–10 days of the surgery. A comparison group consisted of 100 consecutive patients who underwent bilateral removal of indicated mandibular wisdom teeth and did not receive PRF placement within the lower third molar surgical sites. *Results*. The incidence of localized osteitis (LO) following removal of 200 lower third molars with simultaneous PRF placement within the extraction site was 1% (2 sites out of 200). The group of patients whose mandibular 3rd molar sockets were not treated with PRF demonstrated a 9.5% (19 sites out of 200) incidence of localized osteitis. The latter group also required 6.5 hours of additional clinical time to manage LO than the study group who received PRF. *Conclusions*. This retrospective review demonstrated that preventative treatment of localized osteitis can be accomplished using a low cost, autogenous, soluble, biologic material, PRF, that PRF enhanced third-molar socket healing/clot retention and greatly decreased the clinical time required for postoperative management of LO.

## 1. Introduction

Localized osteitis (Dry Sockets) may occur in all locations where teeth are removed, but the majority of localized osteitis develops within the mandibular third-molar region (45% of cases) [1]. Localized osteitis is also called alveolar osteitis, alveolitis sicca dolorosa, septic socket, necrotic socket, localized osteomyelitis, and fibrinolytic alveolitis among other terms to describe this phenomenon [2]. When this condition occurs, it is characterized as postoperative pain surrounding the alveolus that increases in severity during a period of 1–3 days after tooth extraction, followed by partial or complete loss of the initial blood clot in the interior of the alveolus (socket) with or without halitosis [1, 3, 4]. This occurs when initial clot formation fails to mature and the normal socket healing sequence fails [5, 6]. When the clot formation does mature, angioblastic ingrowth occurs through the clot and over the intraoral aspect of the clot, epithelial migration progresses. Fibroplasia of the clot ensues with cellular elimination of fibrin and blood debris, and osteoid formation begins to be generated from locally induced mesenchymal cell activity. Eventually woven bone formation develops through osteoblastic/osteoclastic activity and mature socket bone is finally formed [6]. 

Localized osteitis and its accompanying morbidity are detrimental to patients social/physical well-being and require additional postoperative management compared to patients who do not develop LO. Prevention of LO has been studied over the years and the exact etiology remains elusive but has been associated with trauma during extraction, local anesthetics with vasoconstrictors, oral contraceptive use, gender, patient age, tooth location, smoking, physical dislodgement of the clot, bacterial infection, eruption pattern timing of tooth removal, and operator skill [2, 7, 8]. Preventative treatments have ranged from altered surgical technique, systemic antibiotic use, antimicrobial rinses, socket lavage, to medicaments placed within the socket at the time of surgery (Gelfoam saturated with Cleocin, Gelfoam with Tetracycline, Tetracycline alone, Terra-Cortril on Gelfoam, Chlorhexidine rinses or gel, thorough saline irrigation, use of calcium sulfate and use of activated platelet rich plasma) [2, 7, 9–13]. The application of PRF use within oral and maxillofacial surgery was first described by Dr. Joseph Choukroun, who used a centrifuge to develop a platelet-rich fibrin clot from autogenous whole blood [14]. Dr. Joseph Choukroun of France advocated the use of a platelet concentrate autogenous material (platelet rich fibrin- PRF) in dental extraction sites to expedite socket healing and reduce postoperative pain [15]. The accelerated healing capability of PRF stems from the same growth factors found in another platelet concentrate, platelet-rich plasma (PRP). The technique used to produce PRF also imparts the desirable additive feature of a pliable, suturable fibrin mesh [15]. The use of PRF offers a biologic approach to prevention of LO, expediting healing of the extraction site and in turn decreases postoperative pain and the adverse sequelae subjected to our patients who develop localized osteitis.

## 2. Materials and Methods

We chose the bilateral lower third-molar region to examine the effectiveness of platelet-rich fibrin (PRF) in prevention of LO and clinically based the occurrence of LO on the partial or complete loss of the clot within the third molar site, exposure of the alveolar wall, and regional discomfort in patients treated in our practice. 

### 2.1. Retrospective Review Design

Two hundred patients underwent removal of both mandibular 3rd molars between August 2011 and February 2012 by two surgeons (the authors) in our practice. Each surgeon performed 50 consecutive wisdom teeth removal cases in which PRF was placed in the mandibular third-molar surgical sites. Prior to August 2011, no material was placed in the sockets by either surgeon after removal of mandibular 3rd molars. We used 50 patients treated by each surgeon (total 100 patients) prior to use of PRF for comparison of outcomes as related to development of LO. All patients who received PRF within the lower third molar sites were appropriately consented for application of PRF. The study design was approved by Dr. Dennis J. O'Leary, Chairperson, Florida Internal Review Board Services.

### 2.2. Surgical Procedure

The surgeries were performed under general anesthesia with local anesthesia, and the PRF clot was placed only in the lower third-molar sites bilaterally. A standard buccal flap with disto-buccal releasing incision was instituted when necessary. The blood draws were accomplished following placement of a 22- or 20-gauge angiocatheter, and then a Sureflo injection plug was placed on the angiocatheter. Either a 10 cc syringe or a 21-gauge Terumo blood collection set was used to draw whole blood (8.5–10 cc per Red top BD Vacutainer) captured in 2 different “red top” (noncitrate-containing) sterilized BD Vacutainer blood tubes. The blood tubes were then placed in a standard medical centrifuge for 10–12 minutes at approximately 2700 RPM. The 3rd molars were removed, and the PRF clot was withdrawn from the blood tube using long, thin forceps. Most of the attached red blood cell “tail” was removed from the bottom of the PRF plug and then the PRF plug was placed in each of the mandibular third-molar extraction sites with the platelet-rich (buffy coat) surface directed toward the intra-oral aspect of the socket. The surgical site was closed using 3–0 chromic gut sutures. Primary closure was not always obtained (or attempted) depending on the preexisting location of the lower third molar. The PRF and non-PRF patient groups received preoperative IV antibiotics and steroids (Decadron) as well as postoperative narcotic analgesics (Vicodin/Percocet) and the majority received postoperative oral steroids (Decadron, 4 mg PO Q 8 hours ×6 doses). Both patient groups received standard postoperative instructions, including use of OTC NSAID's in combination with narcotic medications (as needed) and to maintain appropriate oral hygiene with use of OTC antimicrobial mouth rinses for a one-minute rinse at least twice a day. All patients were seen for followup in our practice and accessed for healing progress in addition to presence of localized osteitis within 7–10 days of the surgical procedure.  Table 1 contains patient demographics as well as other details as related to the risk of development of LO. 

Table 2 contains patient demographics and clinical findings for patients who developed localized osteitis.

## 3. Results

Patients experienced postoperative discomfort at the surgical sites which was managed with prescription and nonprescription medications as discussed at the presurgical evaluation. Our practice did not incorporate PRF with mandibular 3rd molar surgery until August, 2011, and the group of patients managed prior to this time frame developed LO in 19 of the 200 sites which equates to an incidence of 9.5%. Only 2 sockets out of the 200 in the group of patients who had PRF placed in the mandibular 3rd molar surgical sites developed LO, an incidence of 1%. In this review of our experience using PRF to prevent LO, we noted that in patients under age 30, their gender, tooth location/difficulty, smoking history, or use of oral contraceptives and steroids did not influence the outcomes. Our limited study did suggest, however, that patient age (age > 30), tooth position/difficulty (complete boney impaction), and systemic disease (insulin-dependent diabetes mellitus) may influence outcomes. 

Less postoperative time was required to manage patients who received PRF. The non-PRF patients averaged 1.47 (147 postoperative visits for 100 patients) postoperative visits while the PRF patients averaged 1.24 (124 postoperative visits for 100 patients) postoperative visits. The thirteen non-PRF patients who developed LO in 19 sites averaged 3.8 postoperative appointments and 395 minutes to manage their LO condition. The two PRF patients who developed LO in two sites averaged two postoperative appointments and 32 minutes to manage their LO condition. The decreased treatment time for management of LO reduced the financial impact of the surgical practice as well as greatly decreased the personal, social, and economic burden subjected to patients who may develop LO following removal of mandibular third molars. The *P* value analysis was used to compare the occurrence of LO in the non-PRF and PRF groups as described in Table 3. Our data demonstrated a very low *P* value which is a strong indicator that PRF helps prevent the formation of LO. A two-sample *z*-test for the difference between proportions was used to compare the occurrence of LO in the Non-PRF and the PRF groups as described in Table 3. It can be concluded that the occurrence of LO is statistically significantly less in the patients who received PRF, (*P* = .0001).

## 4. Discussion 

In our experience of incorporating PRF as a preventive method for development of LO, we found a 90% reduction in the incidence of localized osteitis in patients where PRF was placed within lower third-molar surgical sites and the use of PRF greatly decreased the time required to manage postoperative sequela. 

We believe the reduction of LO stems from retention of the PRF clot by the actions of the fibrin matrix and the degranulation of the platelets. Platelets have been shown to contain alpha granules which upon degranulation release cytokines able to stimulate cell migration and enhance cellular level events to expedite wound healing. These cytokines have been well described and include the following: TGFb-1 (transforming growth factor-beta) is a morphogen that can stimulate osseous cellular activity; PDGF (platelet-derived growth factor) regulates the migration and proliferation of mesenchymal cells in the vicinity of the extraction site to stimulate osseous, endothelial, and fibroblastic proliferation; VGEF (vascular endothelial growth factor) and EGF (epithelial growth factor) aid in the proliferation and differentiation of numerous cell types [15, 16]. Angiogenesis, natural support of immunity, and wound coverage are the keys of soft tissue maturation as described by Choukroun. PRF acts as a natural fibrin-based biomaterial favorable to microvascularization, as a guide to epithelium migration, as well as providing protection of open wounds and accelerates wound healing [15]. The initial organized fibrin matrix of PRF has been described to direct stem cell migration and ideally supports transplanted mesenchymal cells that are essential in directing osseous defect regeneration and seem to be ideal for improving initial third-molar site wound healing, clot stabilization, and prevention of localized osteitis [15–19]. The physical displacement of red blood cells from the socket by placing in PRF and the increased concentration of platelets within the socket also accounts for the enhanced socket-healing capability [11, 12].

Additional investigation of PRF use in the prevention of localized osteitis in mandibular third-molar sites is encouraged to overcome the limitations of our retrospective study in regard to our limited patient data base, lack of a randomized prospective study design, and in some cases, additional teeth were removed along with the lower wisdom teeth and additional prescription of postoperative medications may not have been predicated on symptoms induced solely from removal of the lower third molars. The application of PRF was simple to use and extremely cost effective from an application stand point as well as reduced the clinical time devoted to management of LO, all of which increase the appeal for standard usage during mandibular third-molar surgery. This preventative technique also uses an autogenous, soluble biologic material which does not introduce foreign material into the surgical site and thus, prevents foreign body inflammatory reactions.

## Figures and Tables

**Table 1:** Total study patient clinical data.

Parameter	PRF	Non-PRF
	Patients (*n*)	Patients (*n*)
Age (yrs) ranges		
14–20 years	54	62
21–25 years	23	24
26–30 years	5	8
31–35 years	9	2
35–40 years	4	2
40+ years	5	2
Females	54	41
Males	46	59
Oral contraceptive steroid users	11	8
Smokers/oral tobacco users	5	5
Diabetes (insulin dependent)	1	1
Estrogen therapy (HRT)	1	0
Patients receiving additional narcotic Rx's	15	18
Patients receiving additional steroid Rx's	17	11
PreOperative lower Third-Molar Eval.		
History of pericoronitis	3	5
Fully erupted molar	36	16
Soft tissue impacted molar	6	5
Partially bony impacted molar	84	98
Completely bony impacted molar	71	76

**Table 2:** Clinical data of patients who developed localized osteitis.

Parameter	PRF	Non-PRF
	patients (*n*)	patients (*n*)
Age (yrs) ranges		
14–20 years	0	6
21–25 years	0	4
26–30 years	0	1
31–35 years	1	1
35–40 years	0	0
40+ years	1	1
Females	1	9
Males	1	4
Oral contraceptive steroid users	0	4
Smokers/Oral Tobacco users	0	1
Diabetes (insulin dependent)	1	1
Estrogen therapy (HRT)	1	0
Patients receiving additional narcotic Rx's	2	9
Patients receiving additional steroid Rx's	2	7
Bilaterally occurring localized osteitis	0	6
PreOperative lower third molar eval		
Partially bony impacted molar	0	10
Completely bony impacted molar	2	9

**Table 3:** Statistical analysis.

	LO total (*n*) per		
	200 sites	Incidence (%) of LO	*P* value
PRF group	2	1%	.0001
Non-PRF group	19	9.5%	
